# Comparative Evaluation of Juices from Red-Fleshed Apples after Production with Different Dejuicing Systems and Subsequent Storage

**DOI:** 10.3390/molecules27082459

**Published:** 2022-04-11

**Authors:** Annette Wagner, Stefan Dussling, Stefano Scansani, Peter Bach, Michael Ludwig, Christof B. Steingass, Frank Will, Ralf Schweiggert

**Affiliations:** 1 Workgroup Analysis and Technology of Plant-Based Foods, Department of Beverage Research, Geisenheim University, Von-Lade-Strasse 1, 65366 Geisenheim, Germany; annette.wagner@hs-gm.de (A.W.); stefan.dussling@hs-gm.de (S.D.); peter.bach@hs-gm.de (P.B.); michael.ludwig@hs-gm.de (M.L.); christof.steingass@hs-gm.de (C.B.S.); frank.will@hs-gm.de (F.W.); 2 Department of Microbiology and Biochemistry, Geisenheim University, Von-Lade-Strasse 1, 65366 Geisenheim, Germany; stefano.scansani@hs-gm.de

**Keywords:** spiral filter press, reductive juice processing, vacuum dejuicing, red-fleshed ‘Weirouge’ apples, UHPLC-DAD-ESI-QTOF-HR-MS/MS

## Abstract

In this work, two vintages (2019 and 2020) of red-fleshed ‘Weirouge’ apples were processed with the innovative spiral filter press technology to investigate juice production in an oxygen-reduced atmosphere. After pressing, a more brilliant red color and appreciably higher amounts of oxidation-sensitive constituents (ascorbic acid, anthocyanins, and colorless (poly)phenols) were seen in spiral filter pressed juices compared to those produced with conventional systems (horizontal filter press and decanter). In a subsequent stability study (24 weeks storage at 4, 20, and 37 °C), the color and phenolic compounds were monitored and differences in the juices produced with the different pressing-systems were widely maintained during the storage period. The analyses of the anthocyanins and colorless (poly)phenols were conducted by UHPLC-DAD-ESI-QTOF-HR-MS/MS and UHPLC-DAD. The spiral filter press emerged as a promising technology for the production of juices with a more attractive color and a better retention of oxidation-sensitive constituents during processing and storage compared to conventional juices.

## 1. Introduction

Current scientific evidence found widely undebated correlations between increased fruit and vegetable intake and a lower incidence of metabolic syndrome, cardiovascular disease, cancer, and all-cause mortality [1,2]. Although the underlying molecular mechanisms of the protective effects of fruits and vegetables are still being investigated and controversially debated, the abundance of minerals, vitamins, and dietary fibers in fruits and vegetables appears crucial. Furthermore, antioxidant plant compounds such as carotenoids and (poly)phenols may contribute to such health benefits. For instance, they protect the body against cellular oxidative damage [3] and have been shown to modulate the microbiota of the colon [4,5]. Furthermore, they have been reported to influence human gene expression [6], and to directly interact with receptor and transporter proteins [7]. In addition to their antioxidant properties, (poly)phenols play an important role for characteristics such as taste as well as astringency, color, and flavor perception [8].

Due to their putative health benefits, food processing should aim to retain the levels of the oxidation-sensitive (poly)phenols as much as possible. Concomitantly, diminishing (poly)phenol oxidation is also important to avoid excessive browning of the product, which is generally undesired. With regard to vegetable and fruit juice or puree production, a most common approach to prevent (poly)phenol oxidation is the addition of ascorbic acid or sodium ascorbate [9]. Technological approaches include processing as much as possible under inert atmosphere (e.g., N_2_) as well as implementing vacuum- or membrane-based de-aeration or inert gas sparging [10,11].

A particularly challenging fruit, with regard to oxidation damages during processing, is red-fleshed apple (certain *Malus domestica* Borkh. cvs.) such as cv. ‘Weirouge’, whose vibrant red color is imparted by anthocyanins. The content of anthocyanins in this and thereof derived apple varieties (up to 1203 mg/kg in the peel and 381 mg/kg in the flesh [12]) is much smaller compared to other red fruits such as cherries (up to 2230 mg/kg) or blackberries (up to 1980 mg/kg) [13], and they appear to be less stable during processing and storage [14,15]. Oxidation prevention by adding ascorbate or ascorbic acid should be avoided here because anthocyanins have long been known to be susceptibly degraded by ascorbic acid [16,17,18,19]. 

**Figure 1:**
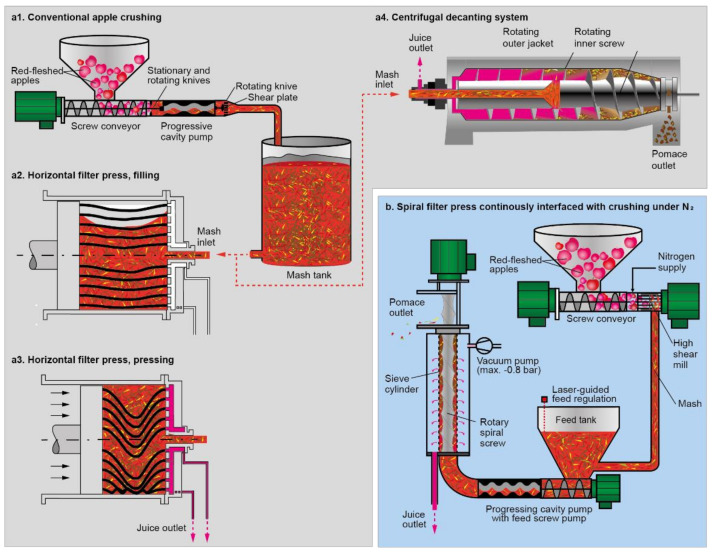
Technological steps during juice processing. (**a**): Conventional crushing (**a1**) and dejuicing with the pressing systems horizontal filter press (**a2**,**a3**) or decanter (**a4**). (**b**): Crushing and dejuicing with the spiral filter press.

The aim of this study was to compare the stability of oxidation-sensitive compounds in red-fleshed apples, such as anthocyanins, during juice extraction with different pressing systems. Therefore, the apples were processed with an innovative vacuum-driven spiral filter press or two conventional processing approaches (hydraulic horizontal filter press and decanter) at pilot plant scale (ca. 200 kg per batch). The spiral filter press has been designed to enable minimal input of oxygen during solid–liquid separation for juice and puree production. After milling under nitrogen gas (N_2_), a progressing cavity pump with feed screw pump transports the mash continuously and rapidly within a closed system to the extraction cell. This cell consists of a sieve cylinder which is placed under reduced pressure and a screw helix (spiral) with mash channels that conveys the mash through the extraction cell. The pressure gradient causes the juice to leave the sieve into an inert atmosphere buffer tank, while the remaining solid part withheld by the sieve is ejected by the screw helix. Concomitantly to solid–liquid separation, the product gets de-aerated by the reduced pressure in the extraction cell. Immediate de-aeration might presumably be highly important despite milling under nitrogen, because substantial amounts of air with up to 14% oxygen [20] are expected to be trapped within the apples’ tissue in intercellular spaces, which have been reported to occupy from ca. 7 to 23% of the total fruit volume [21]. Therefore, besides avoiding oxygen input from the surrounding air, the whole process from milling under N_2_ to dejuicing and de-aeration should be as rapid and continuous as possible without large buffer tanks, if minimizing oxidative damage is the goal. An illustration of the technologies used in this study may be found in Figure 1.

In this study, apples of the variety ‘Weirouge’ of two vintages (2019, 2020) were processed each in technological repetitions at pilot plant scale (ca. 200 kg per batch) to cloudy apple juice using the aforementioned spiral filter-based process as well as with two conventional processes, i.e., a horizontal filter press and a decanter. Subsequently, the juices obtained were stored at 4, 20, and 37 °C for 6 months. Evaluation of the processes and storage stability of the juices was carried out by measuring technology-related parameters such as juice yield, turbidity, viscosity, and the levels of constituents such as anthocyanins, colorless (poly)phenols, and ascorbic acid. The analytical investigations targeted at elucidating qualitative and quantitative compositional changes during industrial-scale juice production in an oxygen-reduced atmosphere and by using conventional processes as well as subsequent storage of the juices. A particular aim was to retain the red color and the phenolic compounds, that may both influence the consumer acceptance.

## 2. Results and Discussion

### 2.1. Juice Parameters after Production

#### 2.1.1. General Parameters

Table 1 shows the physico-chemical parameters of the red apple juices obtained from replicated processing trials (*n* = 2 in both years) with three different pressing systems. Red-fleshed apples of 2019 had a more advanced maturity than those of 2020, ultimately resulting in lower juice yields in 2019 (spiral filter press: ca. 30%; horizontal filter press: 35%; decanter: 29%, cf. Table 1) than in 2020 (66, 70, and 35%, respectively, Table 1), although we had already adjusted the machine settings as described in Materials and Methods. These observations highlight that red-fleshed apples are a rather challenging raw material owing to their poor texture and a short storage life [22]. The loss of texture during maturation appeared to occur faster than that in common white-fleshed apples. Noteworthy, in our trials, the decanter had generally not coped very well with dejuicing the mash from red-fleshed apples, although the exact reasons remain unclear. However, the performance of our discontinuous horizontal filter press was only challenged with the soft raw material in 2019, when long mash residence times in the press and our standard pressing procedures had resulted in cloud contents of 21.3 ± 9.2%, being substantially higher than what had been achieved with the other systems across all trials (1.4–7.7%, Table 1). Consequently, the viscosity of the juices with the high cloud contents was much higher and more variable (354.9 ± 95.5 mPa·s) than those of all other trials (4.7–31.1 mPa·s). In 2020, the viscosities of all juices were lower (4.7–31.1 mPa·s) than in 2019, being a general consequence of the poorer solid–liquid separation performance of all systems due to the raw material of 2019 being softer than that of 2020.

Unlike the cloud content, the turbidity in the juice obtained from the spiral filter press as measured by light scattering was significantly higher (3412–3761 FNU) than in those obtained with the horizontal filter press (1474–2088 FNU) and the decanter (2033–2071 FNU, cf. Table 1). Thus, a particular inverted behavior of cloud content and turbidity of juices from the spiral filter press (‘low’ cloud of 4.6–7.7% but ‘high turbidity’ of 3412–3761 FNU) and those from the horizontal filter press (‘high’ cloud of 7.3–21.1% and ‘low turbidity’ of 1474–2088 FNU) was observed. As the pore size of the filter elements and the principle of solid–liquid separation in the used pressing systems differs, the resulting cloud particles will inherently vary in shape, size, and reflectivity. In the spiral filter press, only particles of a sharp cut-off maximum size of 100 µm are thought to be pulled through the sieve pores, whereas particles of much larger sizes may pass the textile material filter of the horizontal filter press. 

As shown in Table 1, oxygen content after pressing was lowest in juices of the spiral filter press (4.5 ± 0.1 mg/L), followed by those of the horizontal filter press (6.1 ± 1.1 mg/L) and the decanter (10.7 ± 0.0 mg/L). Interestingly, after bottling and cooling back, differences between the oxygen content in the juices produced with different pressing systems were balanced out. In the juice of the spiral filter press, slightly more oxygen was measured (6.6 ± 1.0 mg/L) compared to the, aforementioned, content after pressing. The oxygen level in the juice of the horizontal filter press remained almost constant (6.8 ± 0.4 mg/L), while that of the decanter juice slightly decreased after bottling (7.3 ± 0.3 mg/L). 

It is likely that the processing steps after pressing (i.e., pumping, pasteurizing, and bottling) which were not conducted in an inert atmosphere caused an oxygen intake into the spiral filter pressed juice. The decrease of dissolved oxygen in the decanter juice between pressing and bottling could be a result of oxidation reactions. Independent of the storage temperature, almost all oxygen in the bottled juices (Table 1) was consumed within the first two weeks (Supplementary Table S1). 

Relative density and total soluble solids (TSS, °Brix) were in the same range in both vintages and all pressing systems. They were within the Code of Practice values specified for apple juices by the European Fruit Juice Association (AIJN, rel. density 20/20 of min. 1.0400 g/cm^3^ and TSS of min. 10.0 °Brix). Similarly, sugar contents were widely similar and independent of the processing technology, except for D-glucose, D-fructose, and total sugar contents being slightly higher in 2019′s juices of the spiral filter press (15.0, 65.3, and 114.7 g/L, respectively) than in the others (12.3–12.8, 59.3–59.8, and 103.2–106.6 g/L, respectively). Noteworthy, the lower limit of the AIJN Code of Practice has been laid out at 15–35 g/L for D-glucose for common apple juices, and thus to be considered with care when looking at D-glucose in juices from red-fleshed apples, in our study ranging from 12.3–15.0 g/L. In contrast to glucose, all fructose contents of 2019′s juices were within the range given by the AIJN Code of Practice for common apple juices (45–85 g/L), but sucrose contents in all juices were slightly higher than the upper AIJN limit of 30 g/L (cf. Table 1, 31.8–34.5 g/L). Comparing juices from 2019 and 2020, monosaccharide contents were generally slightly higher, while sucrose amounts were lower in 2020. In this latter vintage, all values were within the AIJN limits and just small differences between the pressing systems were observed (Table 1). 

The citric acid contents in all juices (0.2–0.3 g/L, Table 1) were at the upper limit or slightly higher than that given by the AIJN Code of Practice values (0.05–0.2 g/L). L-malic acid contents (9.9–12.8 g/L, Table 1) were within the AIJN values (min. 3.0 g/L), but comparably high regarding a typical value of 6.0 g/L reported by Eisele et al. [24]. Due to high organic acid amounts, the pH values were 3.1 to 3.3, which is in the lower section of common pH values of apple juice (3.2 to 4.0) [25].

Our findings regarding sugars and organic acids are in agreement with Sadilova et al. [26]. They also found low contents of glucose and fructose and higher contents of sucrose and malic acid compared to literature and suspected a lower invertase activity in ‘Weirouge’ apples. Nevertheless, Ma et al. [27] noted that the contents of sugars and acids depend on genetic and environmental factors. 

#### 2.1.2. Ascorbic Acid and Total Phenols (Folin–Ciocalteu Assay)

A general observation was that the contents in ascorbic acid were ca. 2-fold lower in the more mature apples in 2019 than in those of 2020 (Table 1). Similarly, contents of total phenols were lower in 2019 than 2020 (Table 1), confirming a general difference in the raw materials. More importantly, both contents in ascorbic acid and total phenols were significantly higher in all juices across both vintages made with the spiral filter press (21.0–39.6 mg/L and 795–960 mg/L, respectively) as compared to those made with the other technologies (4.5–10.7 and 405–617 mg/L, respectively, Table 1). In agreement with our findings, Bassi et al. [28] found 30–50 mg/L ascorbic acid in ‘Weirouge’ apples and 5.5 mg/L in juices obtained thereof by belt press, which was comparable to the ascorbic acid contents in the horizontal filter press and decanter juice reported herein. Although the ascorbic acid content in the used fruit has not been analyzed and is anyways highly variable, we believe that spiral filter press production had maintained a high share of the fruit’s genuine ascorbic acid content. Although further study is warranted, the commonly applied addition of ascorbic acid (up to 500 mg/kg [29]) as reducing agent to avoid excessive browning reactions could be redundant. The above-described findings clearly agree with the hypothesis of a significant reduction of oxygen intake by the spiral filter press technology as discussed below in more detail.

#### 2.1.3. Color and Appearance

**Figure 2:**
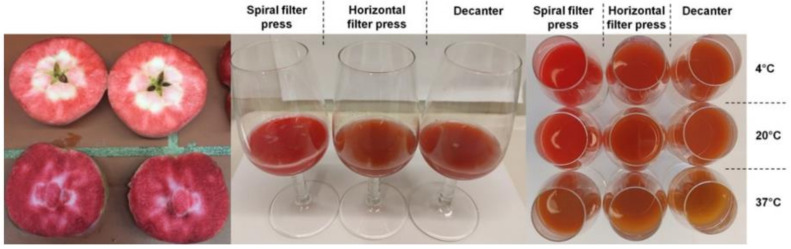
Left: Cross-section of red fleshed apples, cv. ‘Weirouge’. Middle: Raw juice derived of dejuicing red-fleshed apples with three different pressing systems in 2020 (from left to right: spiral filter press, horizontal filter press, and decanter). Right: juices after storage (52 weeks) at three different temperatures (from left to right: spiral filter press, horizontal filter press, and decanter). Top row: 4 °C; middle row: 20 °C; bottom row: 37 °C.

The raw juices produced by spiral filter press had a high color brilliance without browning (Table 1 and Figure 2). The visual color impression was confirmed by significantly higher CIE-a* values in the centrifuged spiral filter press juice (26.3–30.5), lower hue angles (h°: 44.1–44.6), and a higher saturation (C*: 37–42.5) compared to those of the more brownish and less brilliant red juices produced with the conventional technologies (a*: 5.5–13.3; h°: 61.5–67.6; C*: 14.4–31.4) as measured with the spectrophotometer. The CIE-L*a*b* values measured on cloudy juices with the chromameter were in full agreement (Supplementary Table S2).

#### 2.1.4. Anthocyanins

**Figure 3:**
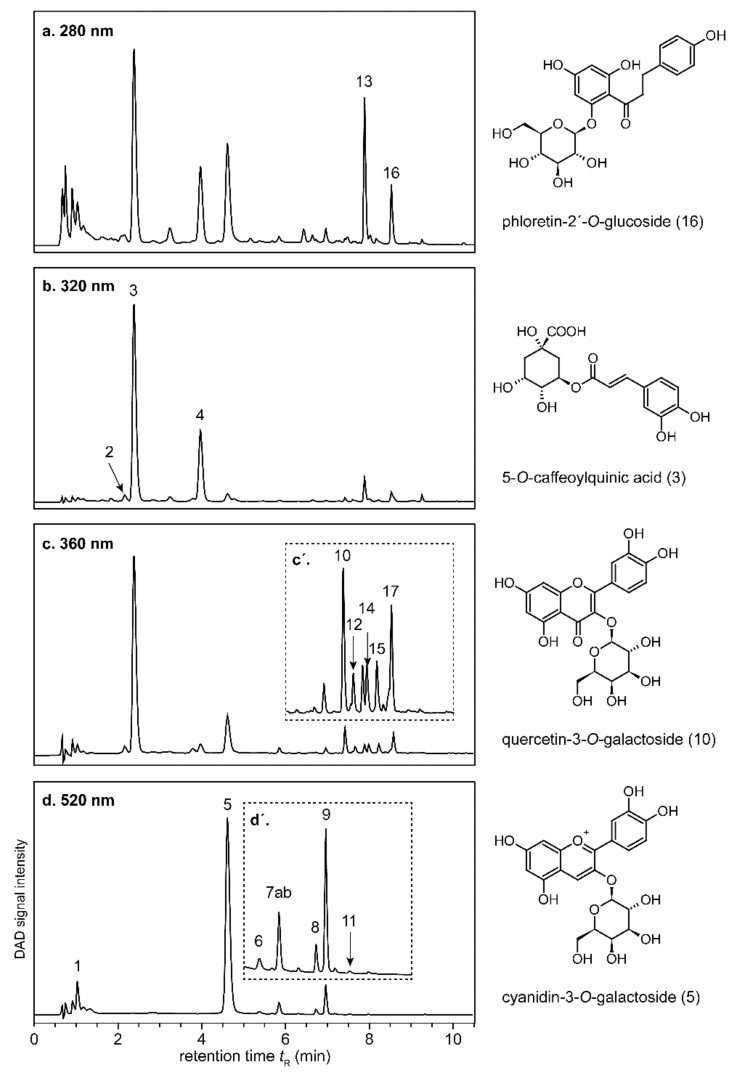
Representative UHPLC-DAD chromatograms of colorless (poly)phenols (**a**–**c**) and anthocyanins (**d**) of a spiral filter pressed juice obtained from red-fleshed ‘Weirouge’ apples. Individual chromatograms are not to scale. The inserts (**c′** and **d′**) represent enlarged sections. For compound assignment, see Table 2.

The aforementioned intense red juice color is known to be caused by anthocyanins, which are commonly found only in the peels of apples, but also in the flesh of the used variety [30,31]. Although the pigments occur widely as comparably stable and red-colored flavylium cation at pH values in apple juice [32], juices from red-fleshed apples suffered from brownish off-colors, hampering their marketability. Table 2 shows the anthocyanins and colorless (poly)phenols analyzed by UHPLC-DAD-ESI-QTOF-HR-MS/MS, the resulting chromatograms are presented in Figure 3. Using the spiral filter press, the freshly pressed and pasteurized juice exerted substantially higher contents of total anthocyanins (47.89–74.91 mg/L) than when using the horizontal filter press or the decanter (12.59–17.26 mg/L) as shown in Table 3. 

**Figure 4:**
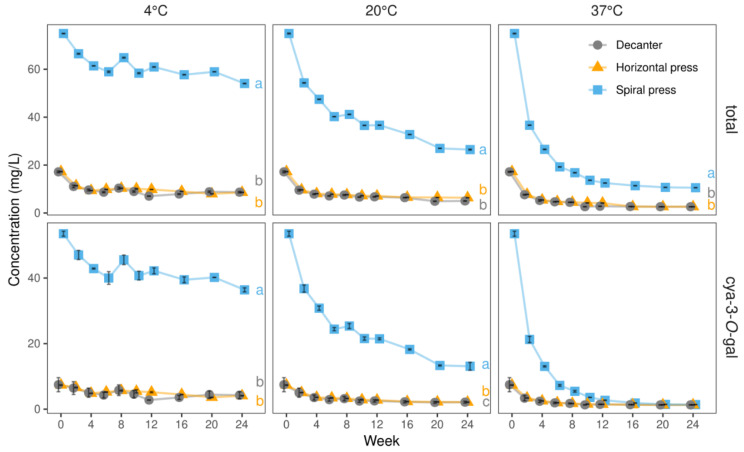
Levels of total anthocyanins and cyanidin-3-*O*-galactoside (cya-3-*O*-gal) in juices derived from three pressing systems in 2020 during 24 weeks of storage at 4, 20, and 37 °C. Data represent means and standard deviations of two technological replicates. Relative anthocyanin degradation was substantially more pronounced in spiral filter pressed juices, owing to their massively higher starting levels. Different letters (a, b) indicate significantly different (*p* < 0.05) means of the respective concentration at the end of the storage study.

The juice richest in anthocyanins (74.91 mg/L) was the 2020 spiral filter press variant, containing 6-fold more than the decanter-made juice in 2019 (12.59 mg/L), which had the lowest amounts. In agreement with Mazza and Velioglu [33] and Su et al. [34], cyanidin-3-*O*-galactoside (cya-3-*O*-gal) prevailed, accounting for 64 and 71% of the total anthocyanins in the spiral filter press-produced juices in 2019 and 2020, respectively. In those obtained by horizontal filter press and decanter, cya-3-*O*-gal merely contributed to 43–49% and 28–43% of the total anthocyanins, respectively. In more detail, the anthocyanin-richest juice (2020, spiral filter press) contained 53.52 mg/L cya-3-*O*-gal, i.e., more than 15-fold than the lowest concentration determined among all juices (3.54 mg/L, 2019, decanter, Table 3). These findings are presumably related to a more advanced oxidative degradation in the decanter and horizontal filter press-produced juices as a considerable loss of cya-3-*O*-gal was observed with progressing storage duration (Figure 4). Interestingly, differences with regard to the remaining anthocyanins were less pronounced when comparing differently produced juices. For instance, contents of cya-3-*O*-glucoside, cya-3-*O*-arabinoside, and cya-3-*O*-pentoside (2) were only up to 1.4-, 1.5-, and 3.8-fold higher when using the spiral filter press as compared to the other technologies (Table 3). 

In a multivariate approach, Malec et al. [15] found oxygen to be the most important factor for color retention in juices made of red-fleshed apples, as juice kept under argon atmosphere had a stronger red and less brownish color than a juice stored under air atmosphere. These findings are in agreement with our observations. However, we believe that spiral filter press processing is not only excluding oxygen entry during processing, but also removes intra- and intercellular oxygen by the dejuicing under reduced pressure (Figure 1). This is particularly relevant for apples, in which air filled spaces can account for 7 to 23% of the total volume of apples [21]. The entire process, i.e., milling, dejuicing, and concomitant removal of oxygen, is completed within 20–30 s. In the time slot between milling and dejuicing, oxidation reactions of the intra- and intercellular oxygen contained in the apple tissue is possible. 

### 2.2. Stability Study

#### 2.2.1. CIE-L*a*b*-Values

**Figure 5:**
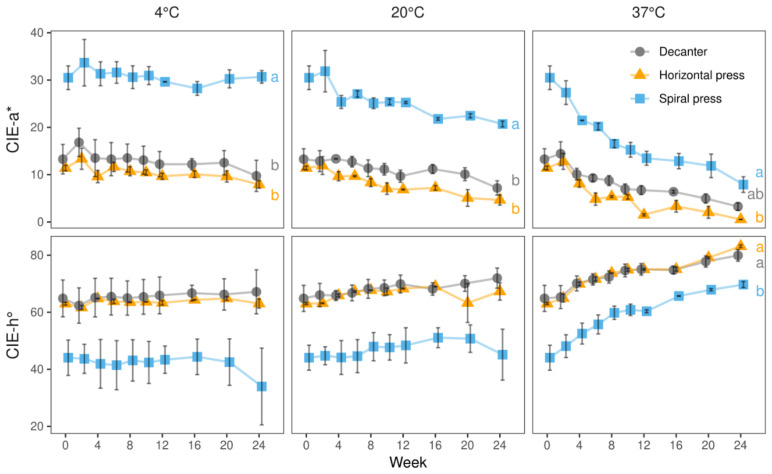
CIE-a* and -h° values of juices derived from three pressing systems during 24 weeks of storage at 4, 20, and 37 °C in 2020. Data represent means and standard deviations of two technological replicates. The substantially more reddish tonality of juices produced with spiral filter press was maintained throughout the full storage period at 4 °C. Although a minor and major degradation was observed at 20 and 37 °C, the color difference between spiral filter press-produced juices and those made with the other technologies was sustained. Different letters (a, b) indicate significantly different (*p* < 0.05) means of the respective color value at the end of the storage study.

At 4 °C, the redness (CIE-a*) and color hue (CIE-h°) of the spiral filter press-produced juices remained constant throughout 24 weeks of storage (Figure 5). In contrast, the CIE-a* values of the juices made with the horizontal filter press and the decanter had not only started at substantially lower values, but also decreased slightly during storage. For instance, CIE-a* decreased from ca. 11.4 and 13.3 to ca. 7.9 and 9.8 in horizontal filter press- and decanter-made juices, respectively. The hue angles just slightly changed during storage at 4 °C (Figure 5). 

At 20 °C, slightly declining CIE-a* values and increasing hue angles were seen irrespective of the pressing systems. CIE-a* values of spiral filter press-produced juices decreased during storage from ca. 30.5 to 20.7 whereas those of the juices made with the horizontal filter press and the decanter decreased from 11.4 to 4.7 and from 13.3 to 7.2, respectively. Concomitantly, an increase in hue angle was observed in all juices as shown in Figure 5. Our results indicate that after 24 weeks of storage, the red color of the spiral filter pressed juice had been more intense and with less brownish hues than that of the juices obtained by conventional processing. 

At 37 °C, the change of color was most striking and a strong shift towards brown was seen in all juices. Noteworthy, although spiral filter pressed juices changed their colors more drastically than the others, its final color was still characterized by a substantially more reddish tonality (Figure 5). 

#### 2.2.2. Anthocyanins

During storage at 4 °C, the total anthocyanin contents just slightly declined, i.e., from 74.9 to 54.1 mg/L, from 17.3 to 8.6 mg/L, and from 17.2 to 8.7 mg/L after spiral filter press, horizontal filter press, and decanter dejuicing, respectively. The total anthocyanins were more stable in the spiral filter pressed juice, as the percentages of retained total anthocyanins after 24 weeks (72%) was higher compared to the reference juices (50% and 51% for horizontal filter press and decanter, respectively). The losses were majorly caused by a degradation of the main pigment cya-3-*O*-gal, decreasing from 53.5 to 36.4 mg/L (equaling a retention of 68%) after 24 weeks in spiral filter pressed juice and from 7.4 to 4.1 mg/L (56%) and 7.5 to 4.2 mg/L (57%) in horizontal filter press- and decanter-made juices, respectively. 

At 20 °C, total anthocyanin contents declined continuously during storage with the sharpest drop in the first two weeks. In the spiral filter pressed juices, the half-life (*t*_1/2_) was reached after ca. 10 weeks, while after 24 weeks only 35% of the initial content remained. In the juices made with the horizontal filter press, *t*_1/2_ was four weeks, and thereafter, virtually no relevant further changes were seen. In the decanter juices, *t*_1/2_ was ca. three weeks, with 29% of the initial amounts remaining after 24 weeks. Despite the drastic initial decrease, spiral filter pressed juices still contained higher total anthocyanin levels after 24 weeks at 20 °C than those determined in the remaining juices immediately after production, agreeing with our visual observations and CIE-L*a*b* measurements. 

The stability of the main anthocyanin cya-3-*O*-gal was lower in the spiral filter pressed juice (*t*_1/2_ = six weeks, 25% left after 24 weeks at 20 °C) compared to that in the juices made with horizontal filter press and decanter (both *t*_1/2_ = four weeks and 29% left after 24 weeks at 20 °C). 

During storage at 37 °C, *t*_1/2_ of total anthocyanins was less than two weeks in all juices. At the end of storage, 10.6 mg/L total anthocyanins were found in the spiral filter pressed juice compared to 2.6 mg/L in the juices produced by horizontal filter press and decanter. Almost identical contents of cya-3-*O*-gal (1.3 mg/L) were found in all juices at the end of the storage trial. 

Knebel et al. [35] have previously found lower *t*_1/2_ for total anthocyanins (two weeks) in juices produced from red-fleshed ‘Maggie’ apples applying a horizontal filter press stored at 4 and 20 °C for one year. However, the 200 mg/L of ascorbic acid admixed after pressing may have resulted in a rather low anthocyanin stability. Similarly, Farr and Giusti [16] have reported anthocyanin degradation and extensive bleaching after admixture of 250–1000 mg/L ascorbic acid to chokeberry extracts and cya-3-*O*-gal model solutions, the latter representing the prevailing pigment of red-fleshed apples (Figure 3). In agreement, the concomitant decrease in total anthocyanins and ascorbic acid in the spiral filter pressed juice that had contained higher initial contents of both compounds might be explained by their mutual breakdown reactions.

#### 2.2.3. Colorless (Poly)phenols

The main phenolic constituents of red-fleshed apples have been previously reported [12,15], and, thus, we refer to Table 2 for their identification by UHPLC-DAD-ESI-QTOF-HR-MS/MS without discussion herein.

The most abundant phenolic compound in the freshly produced juices was 5-*O*-caffeoylquinic acid (Table 3), being found in 2020 at almost 3-fold higher levels in juices made with the spiral filter press (150.62 mg/L) compared to those made with the horizontal filter press (49.17 mg/L) and the decanter (57.34 mg/L). The second most abundant constituent was the dihydrochalcone phloretin-2′-*O*-xyloglucoside whose levels in the spiral filter press juices (73.43 mg/L) outperformed those in the horizontal filter pressed (26.36 mg/L) and the decanter juices (34.91 mg/L). Similarly, phloretin-2′-*O*-glucoside contents were 21.74 mg/L in the spiral filter pressed juices, whereas only 12.01 mg/L were found in in the horizontal filter pressed and 10.65 mg/L in the decanter juices. In agreement with our findings, 5-*O*-caffeoylquinic acid has been previously found to be the main phenolic compound in red-fleshed apples [32,35,36]. Phloretin-2′-*O*-xyloglucoside has been reported to be another prevailing colorless (poly)phenol in apples [36], being even more abundant in red-fleshed than in white-fleshed varieties [12]. 

**Figure 6:**
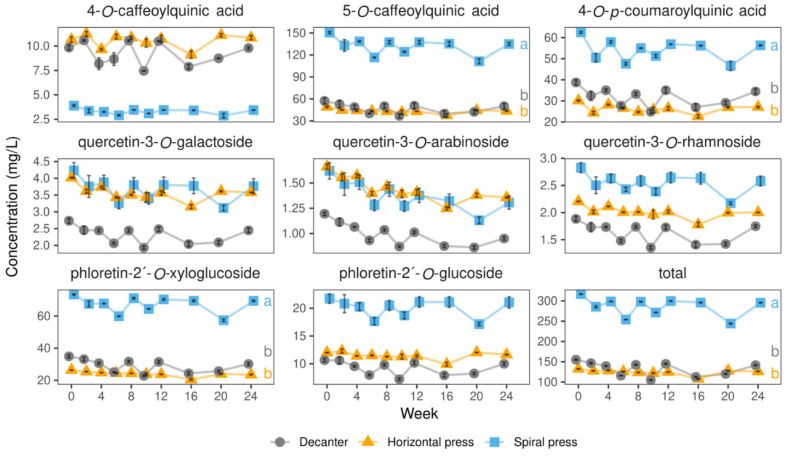
Levels of colorless (poly)phenols in juices derived from three pressing systems in 2020 during 24 weeks of storage at 20 °C. Data represent means and standard deviations of two technological replicates. Relative concentrations of most phenolic compounds monitored were almost stable over time. Different letters (a, b) indicate significantly different (*p* < 0.05) means of the respective color value at the end of the storage study.

In contrast to 5-*O*-caffeoylquinic acid and the phloretin derivatives, the levels of 4-*O*-caffeoylquinic acid were ca. 2-fold lower in spiral filter pressed juices (3.90 mg/L) than in those made with the other technologies (9.84–10.65 mg/L, Figure 6). Quercetin-3-*O*-galactoside (2.73–4.23 mg/L) was the most prominent flavonol in all varieties, which is in accordance with a previous report by [37]. Furthermore, the amounts of all quercetin derivatives were higher in the spiral filter press-made and horizontal filter press-made juices (quercetin-3-*O*-arabinoside, quercetin-3-*O*-galactoside, and quercetin-3-*O*-rhamnoside: 1.62–1.66, 4.02–4.23, and 2.21–2.83 mg/L, respectively) compared to those made with the decanter (1.20, 2.73, and 1.88 mg/L, respectively, Figure 6). 

These findings may be explained by the higher amounts of quercetin derivatives in the skin [15] and the assumption that such skin particles presumably represent part of the increased turbidity in the spiral and horizontal filter pressed juices (Table 1). 

Figure 6 exemplarily shows the stability of the colorless (poly)phenols during storage at 20 °C. The data for storage at 4 and 37 °C is in the Supplementary Tables S3 and S4. Generally, the stability of most colorless (poly)phenols was superior to those of the anthocyanins and was comparable for all compounds assessed (Figure 6). We also noticed rather high fluctuations in the levels of colorless (poly)phenols, which is possibly related to the fact that (poly)phenols are often bound to the cloud whose contents varied in each bottle. The retention of total colorless (poly)phenols was high 91–95% during 24 weeks of storage at 4 and 20 °C and 75–77% at 37 °C. These findings agree with the results of Knebel et al. [35] who found 91–99% of the initial levels after 52 weeks of storage at 4 and 20 °C and 78–91% at 37 °C. However, as noted above, Knebel et al. [35] had added 200 mg/L ascorbic acid being well known to stabilize colorless (poly)phenols.

#### 2.2.4. Ascorbic Acid, Total Phenols (Folin–Ciocalteu Assay), and Antioxidant Capacity (TEAC Assay)

**Figure 7:**
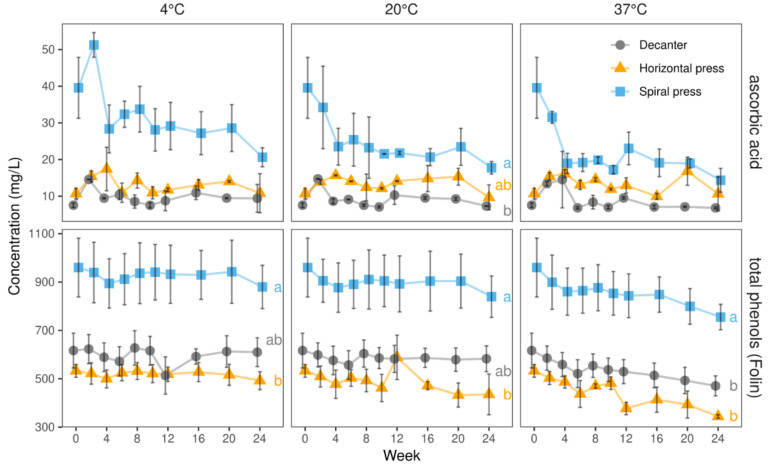
Ascorbic acid and total phenols (Folin–Ciocalteu assay) in juices derived from three pressing systems during 24 weeks of storage at three temperatures (4, 20, and 37 °C) in 2020. Data represent means and standard deviations of two technological replicates. Relative ascorbic acid degradation was substantially more pronounced in spiral filter pressed juices, owing to their higher starting levels. Total phenols were relatively stable regardless of the temperature. Different letters (a, b) indicate significantly different (*p* < 0.05) means of the respective color value at the end of the storage study.

As mentioned above, spiral filter pressed juices contained substantially more ascorbic acid and total phenols than those made with horizontal filter press and decanter (Table 1, Figure 7). At the end of the storage period at 4, 20, and 37 °C, spiral filter pressed juices contained 52, 45, and 36%, respectively, of their initial ascorbic acid levels. Independent of the temperature, the strongest decline was seen in the first four weeks of storage. After 24 weeks, the levels still exceeded those in the juices made with horizontal filter press and decanter. Ascorbic acid levels in the latter two juices were not only smaller but also were more stable during storage.

In agreement with our UHPLC results (Figure 6), total phenol measurements confirmed the high stability during 24 weeks of storage at three different temperatures. Especially at 4 °C, the initial contents (Table 1) were well retained with more than 90% remaining after 24 weeks in all juices. At 20 °C, more than 80% of the initial total phenols were found after 24 weeks. At 37 °C, still 79% remained in the spiral filter pressed, 64% in the horizontal filter pressed, and 76% in the decanter-made juices, corresponding to a loss of 205, 189, and 146 mg/L, respectively.

A strong correlation between the total phenols and the antioxidant capacity (TEAC values) was seen at all temperatures. After 24 weeks of storage, the antioxidative capacities of the juices produced with the single pressing systems at different temperatures were similar, with slightly lower contents in the juice stored at 37 °C. By analogy to the total phenols, highest values were seen in the spiral filter pressed juices, while the decanter juices had a slightly lower capacity being still higher than that found in the horizontal filter press-made juices (cf. Supplementary Table S5).

## 3. Materials and Methods

### 3.1. Production of Cloudy Red-Fleshed Apple Juice

A total of each 1500 kg red-fleshed apples (*Malus domestica* Borkh. cv. ‘Weirouge‘) was purchased at a commercial apple producer (Bleichhof, Meckenheim, Germany) in October 2019 and September 2020, respectively. Batch size was ca. 200 kg for each one of two technical repetitions per year, irrespective of the used processing technology (Figure 1). Noteworthy, the apples of 2019 were softer and at more advanced maturity than those of 2020, which required slightly different setups of the processing technologies as described below. 

The spiral filter press juices were produced with a VaculiQ-1000 (VaculiQ, Hamminkeln, Germany) device. First, the apples were crushed by the integrated mill (MultiCut-unit), which was run at 50 Hz with 3–5 L/min nitrogen. In 2019, a spiral with three mash channels was used as well as a sieve with a pore size of 100 µm. Due to the harder fruit tissue, a spiral with four mash channels was used for processing in 2020. The spiral speed regulator was set at 100 Hz in all productions. The applied vacuum was 0.1 bar below atmospheric pressure in 2019. In 2020, a vacuum of 0.8 bar below atmospheric pressure was used. After spiral filter dejuicing, the raw juice was collected in an inert atmosphere (N_2_) buffer tank.

For reference juice productions with horizontal filter press (HP-L 200, Bucher, Niederweningen, Switzerland) and decanter (Z23-3, Flottweg, Vilsbiburg, Germany), the apples were crushed by a progressive cavity pump with an extended compression casing with an integrated cutting mechanism (open hopper pump BTM Seepex, Bottrop, Germany), targeting a grinding degree of 10 mm by selecting a shear plate with the corresponding pore size (cf. Figure 1). The horizontal filter press was initially filled with 40 kg mesh. By adding mash in 20 kg steps every two minutes after every press holding time (press cycle duration), 200 kg were pressed in total per batch. Total pressing time was 60 min.

For decanter dejuicing, rotational speed of the outer jacket was set at 5200 rpm and the inner screw was adjusted to reach a differential speed of 10–13 U/min. The diameter of the weir plate was 145–150 mm. The raw juice of the reference technologies was collected in a buffer tank without inert atmosphere according to conventional practice. All raw juices were rapidly heated to ca. 78 °C with a fruit juice dispenser (PAS1-PS2-81-V2, Mabo, Eppingen, Germany), and, subsequently, hot-filled into amber glass bottles of 0.33 L volume. Cooling from 78 to 20 °C was achieved in ca. 15 min. The temperature–time profile was recorded and was equivalent to a total pasteurization value (*P*-value) of ca. 2.5, using a *z*-value of 10 K and a reference temperature *T_ref_* of 80 °C according to Equation (1). For the storage stability study, the above-mentioned 0.33 L-bottles were subjected to three different temperatures (4, 20, and 37 °C) in a dark environment. Juice from an individual bottle per time point was taken at nine different sampling points over 6 months and frozen at −20 °C until further analyses.

(1) $$P=\overset{n}{\underset{i}{\sum }}L_{i}=\overset{n}{\underset{i}{\sum }}(t_{i}\times 10^{\frac{T_{i}-T_{ref}}{z}})$$

where *P* is the pasteurization value, *L_i_* the lethal effect of the time interval *i* of a total of *n* time intervals, *t_i_* the duration of the time interval in min, *T_i_* the minimum temperature of the time interval, *T_ref_* the reference temperature (80 °C), and *z* the corresponding *z*-value (10 K). 

### 3.2. Measurement of Dissolved Oxygen in the Juice

For determination of oxygen dissolved in the juice, a galvanic oxygen probe coupled with a portable instrument (probe CellOx 325 with instrument Oxi 3310, WTW, Weilheim, Germany) was used. Immediately after dejuicing, an aliquot of 500 mL of freshly pressed juice was taken from the storage tank to immediately measure oxygen content considering the simultaneously measured temperature. In the storage stability study, the bottles were first brought to room temperature prior to oxygen measurement immediately after opening the bottles. For the storage stability study, measurements took place one day after juice production and at nine dates in six months of storage. During all measurements, the sample was stirred by a magnetic stirrer. 

### 3.3. Determination of Physico-Chemical Parameters

Relative density was measured with a flexural resometer (DMA 48, Anton Paar, Graz, Austria) on the basis of the IFU-method No. 1 [38]. A digital refractometer (Abbémat, Dr. Kernchen, Seelze, Germany) was used to measure the total soluble solids (TSS, °Brix) at 20 °C. The extract content was taken from the density table of Reichard [39]. The non-sugar extract was determined refractometrically based on IFU-method No. 8 [38], and calculated as extract content minus reducing sugars after inversion. Individual sugars (D-glucose, D-fructose, sucrose), citric acid and L-malic acid were determined enzymatically (IFU-methods No. 55, 56, 22 and 21, respectively [38]). Ascorbic acid was determined by iodometry [40]. The pH-value was measured potentiometrically at 20 °C (IFU-method No. 11 [38]). Total acidity (expressed as citric acid at pH 8.1) was titrated (Titrator, Schott, Mainz, Germany) based on IFU-method No. 3 [38]. The amount of total phenols was determined spectrophotometrically with the Folin–Ciocalteu reagent according to [41]. Catechin was used as reference. TEAC [mmol/L Trolox eq.] was determined by spectrophotometry (UVmini-1240, Shimadzu, Suzhou, China) according to Re et al. [42]. Centrifugable cloud was determined by centrifuging 10 g of juice for 15 min at 4200× *g*. After decanting the supernatant, the centrifuge tube was invertedly placed on the lab bench to allow dripping off residual liquid for 30 min at room temperature prior to gravimetrically determining the cloud content in % from the weight of the pellet. For the measurement of turbidity, a turbidity photometer (Nephla LPG 239.52, Dr. Bruno Lange, Berlin, Germany) was used. 

### 3.4. Determination of Objective Color Values (CIE-L*a*b*)

CIE-L*a*b* color values of cloudy raw juice samples were determined with a chromameter (CR-300, Konica Minolta, Osaka, Japan) and a spectrophotometer (Unicam 500, Thermo Electron, Dreieich, Germany). While cloudy samples were measured with the aforementioned chromameter, we eliminated the influence of the cloud prior to color measurement by spectrophotometry by centrifuging the juice samples (5 min at 12,850× *g*). Then, aliquots of 5 mL of sample were used in continuous flow in a 2 mm cuvette (Hellma, Müllheim, Germany). The resulting CIE-L*a*b* values given by the software Vision Pro V 2.03 (Thermo Electron) were used to calculate the color saturation (chroma C* = (a*^2^ + b*^2^)^1/2^) and color hue angle (h° = arctan (b*/a*)). 

### 3.5. Rheology

Rheological behavior was determined with a rotational viscometer MCR 92 (Anton Paar, Graz, Austria) equipped with a temperature control device MCR 92. A cone-plate measuring system (diameter 49.95 mm, cone angle 0.99°) was used to measure viscosity and shear stress. For each measurement, aliquots of 0.6 mL raw juice were tempered to 20 °C and 20 measuring points were recorded in a shear rate range of 0.1 to 100 1/s. Each sample was measured in duplicate. The Rheoplus V3.4 software (Anton Paar) was used for data processing. 

### 3.6. Identification and Quantification of Anthocyanins and Colorless (Poly)phenols

For simultaneous UHPLC analyses of anthocyanins and colorless (poly)phenols, 15 mL of juice were centrifuged (10 min at 12,850× *g*). An aliquot of 7.5 mL of the supernatant was combined with 2.5 mL of methanol following centrifugation for removal of alcohol-insoluble solids such as pectin. The resulting supernatant was filtered with a syringe filter of 0.45 µm pore size (glass fiber pre-filter, reg. cellulose) and transferred into amber HPLC vials. Quantification was carried out applying an UHPLC-DAD system (UltiMate 3000, Thermo Fisher, Waltham, MA, USA) equipped with a C_18_-column (Luna Omega, 100 × 2.1 mm, 1.6 μm particle size, Phenomenex, Aschaffenburg, Germany) with a guard cartridge of the same material. Injection volume was 2 µL, the flow rate was 350 µL/min at 40 °C. Solvent A was water/formic acid (90/10 *v*/*v*), and solvent B was acetonitrile/formic acid (90/10 *v*/*v*). The gradient program with a total run time of 13.5 min was: isocratic hold at 5% B (1 min), 5 to 15% B (3.5 min), 15 to 27.5% B (3.5 min), 27.5 to 50% B (1.5 min), isocratic hold at 50% B (1 min), 50 to 5% B (0.5 min) and isocratic hold at 5% B (2.5 min). For the quantification of single anthocyanins, a linear calibration curve was set up with an authentic cyanidin-3-*O*-galactoside (Extrasynthèse, Genay, France). For quantification of colorless (poly)phenols, standard substances (phloretin-2′-*O*-xyloglucoside, quercetin-3-*O*-xyloside (TransMIT Chemicals Shop, Gießen, Germany), 4-*O*-caffeoylquinic acid, 5-*O*-caffeoylquinic acid, *p*-coumaric acid (Sigma Aldrich, St. Louis, MO, USA), quercetin-3-*O*-rutinoside, quercetin-3-*O*-galactoside, quercetin-3-*O*-arabinoside, quercetin-3-*O*-rhamnoside, and phloretin-2′-*O*-glucoside (Extrasynthèse)) were used for external calibration. Flavanols and dihydrochalcones were quantitated at a wavelength of 280 nm, phenolic acids at 320 nm, and flavonols at 360 nm. 

UHPLC-DAD-ESI-QTOF-HR-MS/MS experiments were performed applying an Elute SP UHPLC system and a *tims*TOF mass spectrometer with an electrospray ionization (ESI) source. The system was operated and data analyzed by applying Compass HyStar 5.1, Compass otofControl 6.0, and Compass Data Analysis 5.3 software (all from Bruker Daltonik, Bremen, Germany). The column, eluents, and UHPLC parameters were as detailed above. Mass spectrometer settings were as follows: scan range, *m/z* 50–1350; spectral rate, 10 Hz; capillary potential 3500 and 4200 V for the negative and positive ion mode, respectively; nebulizing gas pressure, 3.0 bar (N_2_); dry gas flow rate, 7 L/min (N_2_); nebulizer temperature, 250 °C; collision energy stepping, 20–50 eV. The mass spectrometer was calibrated using sodium formate cluster ions.

### 3.7. Statistics

This work compares the production of apple juice using three different pressing systems, namely a spiral filter press, a horizontal filter press, and a decanter. Juices were produced in two technological replicates per year in two separate years (*n* = 2 per year, *n* = 4 in total), using annually available raw materials. Juices obtained in the 2020 production were monitored in a subsequent stability study where the impact of time and temperature on stored juice was studied. Samples were analyzed on nine dates, every two weeks from production through 12 weeks and then every four weeks through 24 weeks and the conclusion of the storage trial. The study temperatures were 4, 20, and 37 °C. Analytical measurement of juices was conducted at each production and storage time in two analytical replicates. The mean and standard deviation (SD) of each analyte were calculated and presented as mean ± SD. Analysis of variance (ANOVA) was performed (α = 0.05) comparing data from the three different pressing systems within each year, followed by the Tukey’s HSD test if statistical significance was indicated by the ANOVA. Statistical analyses and graphs were calculated using base R (version 4.0.2—R Core Team, 2020), the *agricolae* library (de Mendiburu and Yaseen, 2020), and the *tidyverse* package (Wickham et al., 2019).

## 4. Conclusions

In brief, the production of fruit juice from red-fleshed apples with the vacuum-driven spiral filter press permitted to retain not only an attractive color but also high levels of nutritionally favorable constituents. Due to a fast and continuous production in an atmosphere low in oxygen as well as the immediate vacuum-driven de-aeration, the oxygen amounts after pressing were significantly lower in the spiral filter pressed juice compared to those in horizontal filter press- and decanter-made juices. As a consequence, substantially higher levels of anthocyanins and colorless (poly)phenols, ascorbic acid, total phenolics, and an elevated antioxidant capacity was found in the spiral filter pressed samples. Owing to their high initial levels, the anthocyanin-based reddish color was maintained for six months at 4 and 20 °C. Even after 12 months, the color was more reddish in spiral filter pressed juices than in those made with the other two technologies. In our study, the ambivalent role of ascorbic acid also became evident, especially by comparing our results to those in literature. Thereby, in juices of red-fleshed apples, ascorbic acid seems to promote retention of the colorless (poly)phenols but also to accelerate anthocyanin degradation. Finally, we noticed that, for industrial-scale juice production, the throughput volume of spiral filter presses may represent a limiting factor (up to 3 t/h) compared to that of other continuous pressing systems such as decanters (5–10 t/h). Furthermore, operating the spiral filter press technology requires better trained and more intense personnel resources, being possibly challenging when aiming at simultaneously operating several systems. Our experience in juice production with the spiral filter press showed that process monitoring was more elaborate compared to horizontal filter press and decanter, particularly as changing raw material characteristics required frequent adjustments in machine settings. Still, using this innovative processing technology permitted the retention of constituents susceptible to oxidation and thus, juices with superior nutritional value compared to that of conventionally produced juices. Future research may assess the applicability of this technology to process fruits and vegetables other than apples and may additionally focus on its impact on volatile aroma compounds and the sensory quality of the obtained products.

## Figures and Tables

**Table 1:** Physico-chemical parameters and CIE-L*a*b* values of raw juice derived from dejuicing red-fleshed apples with three different pressing systems in 2019 (*n* = 2, technological replicates) and 2020 (*n* = 2).

	2019				2020	
	Spiral Filter Press	Horizotal Filter Press	Decanter	Spiral Filter Press	Horizotal Filter Press	Decanter
yield [%]	30.3 ± 1.8	35.3 ± 2.5	29.0 ± 2.8	66.3 ± 5.0 ^a^	70.1 ± 9.0 ^a^	35.3 ± 1.1 ^b^
cloud content [%]	7.7 ± 1.5	21.3 ± 9.2	3.8 ± 1.2	4.6 ± 0.4	7.3 ± 5	1.4 ± 0.3
turbidity [FNU]	3412 ± 286 ^a^	2088 ± 228 ^b^	2033 ± 50 ^b^	3761 ± 349 ^a^	1474 ± 204 ^b^	2071 ± 143 ^b^
viscosity [mPa·s] ^5^	27.8 ± 1.3 ^1^	354.9 ± 95.5 ^1^	5.8 ± 0.5 ^1^	5.8 ± 1.9	31.1 ± 10.4	4.7 ± 1.8
oxygen [mg/L] - after pressing	n.a. ^2^	n.a. ^2^	n.a. ^2^	4.5 ± 0.1 ^b^	6.1 ± 1.1 ^b^	10.7 ± 0.0 ^a^
- after bottling	n.a. ^2^	n.a. ^2^	n.a. ^2^	6.6 ± 1.0	6.8 ± 0.4	7.3 ± 0.3
density d(20/20) (g/mL)	1.0544 ± 0.0009	1.0510 ± 0.0003	1.0491 ± 0.0025	1.0563 ± 0.0024	1.0555 ± 0.0008	1.0551 ± 0.0004
TSS (°Brix)	13.1 ± 0.2	12.3 ± 0.1	11.8 ± 0.6	13.5 ± 0.6	13.3 ± 0.2	13.2 ± 0.1
extract [g/L]	141.4 ± 2.4	132.4 ± 0.7	127.5 ± 6.6	146.4 ± 6.3	144.2 ± 2	143.0 ± 1.0
pH value	3.3 ± 0	3.3 ± 0	3.3 ± 0	3.1 ± 0	3.2 ± 0.1	3.1 ± 0
total sugar [g/L]	114.7 ± 3.3	106.6 ± 1.6	103.2 ± 5.1	118.8 ± 5.6	117.8 ± 1.7	117.9 ± 1.3
D-glucose [g/L]	15.0 ± 0.4 ^a^	12.8 ± 0.6 ^ab^	12.3 ± 0.8 ^b^	18.7 ± 1.9	18.4 ± 1.5	18.8 ± 0.1
D-fructose [g/L]	65.3 ± 1.6	59.8 ± 0.8	59.3 ± 2.5	70.5 ± 2.7	69.9 ± 1	72.7 ± 1.8
sucrose [g/L]	34.5 ± 1.2	34.1 ± 0.1	31.8 ± 1.9	29.6 ± 1.0 ^a^	29.5 ± 0.8 ^a^	26.5 ± 0.5 ^b^
total acidity [g/L]	7.8 ± 0.1	7.3 ± 0.4	7.1 ± 0.3	10.3 ± 0.4	9.1 ± 0.8	9.1 ± 0.1
citric acid [g/L]	0.2 ± 0	0.2 ± 0	0.2 ± 0	0.3 ± 0 ^a^	0.2 ± 0 ^ab^	0.2 ± 0 ^b^
L-malic acid [g/L]	10.5 ± 0.2	10.2 ± 0.8	9.9 ± 0.5	12.8 ± 0.4	11.3 ± 1.0	11.3 ± 0.1
ascorbic acid [mg/L]	21.0 ± 2.8 ^a^	5.5 ± 0.7 ^b^	4.5 ± 0.7 ^b^	39.6 ± 8.3 ^a^	10.7 ± 1.5 ^b^	7.5 ± 0.6 ^b^
total phenols [mg/L] ^3^	795 ± 7 ^a^	405 ± 11 ^b^	425 ± 10 ^b^	960 ± 122 ^a^	533 ± 27 ^b^	617 ± 72 ^ab^
antioxidant capacity (mmol/L) ^4^	6.2 ± 0.7 ^a^	3.2 ± 0.3 ^b^	3.4 ± 0.1 ^b^	6.9 ± 0.6 ^a^	3.9 ± 0.3 ^b^	4.4 ± 0.5 ^b^
CIE-L*a*b*						
L*	58.4 ± 7.1 ^b^	85.9 ± 1 ^a^	88.1 ± 1.3 ^a^	41.7 ± 13.2	67.9 ± 5.7	58.1 ± 0.9
a*	26.3 ± 0.6 ^a^	7.4 ± 0.1 ^b^	5.5 ± 0.9 ^b^	30.5 ± 3.5 ^a^	11.4 ± 0.6 ^b^	13.3 ± 3.1 ^b^
b*	26.0 ± 3.0 ^a^	13.6 ± 1.2 ^b^	13.3 ± 0.5 ^b^	29.5 ± 3.0	22.4 ± 1.7	28.3 ± 1.7
hue angle h°	44.6 ± 3.9 ^b^	61.5 ± 2.3 ^a^	67.6 ± 2.4 ^a^	44.1 ± 6.2 ^b^	63.0 ± 0.5 ^a^	64.9 ± 6.5 ^a^
chroma C*	37.0 ± 1.7 ^a^	15.5 ± 1 ^b^	14.4 ± 0.8 ^b^	42.5 ± 0.5 ^a^	25.1 ± 1.7 ^c^	31.4 ± 0.2 ^b^

^1^: please note the higher viscosity caused by slightly overripe and, thus, soft raw materials in 2019 as affected differently by the pressing systems. ^2^: oxygen measurements were unavailable in 2019. ^3^: as measured by the Folin–Ciocalteu assay. ^4^: as measured by Trolox equivalent antioxidant capacity (TEAC) assay. ^5^: as measured at a shear rate of 1/50 s, mimicking the oral shear stress for liquids according to [23]. TE: Trolox equivalents. TSS: total soluble solids. Different superscript letters (a, b, c) indicate significant (*p* < 0.05) differences of means within one year.

**Table 2:** UHPLC-DAD-ESI-QTOF-HR-MS/MS analysis of anthocyanins and colorless phenolic compounds in juices from red-fleshed apples.

No.	Proposed Structure	*t*_R_ (min)	UV/vis λ_max_ (nm)	Precursor	Exp. *m/z*	Calc. *m/z*	Error (ppm)	Formula (Precursor)	QTOF-HR-MS/MS *m/z* (% Base Peak Intensity)
1	anthocyanin-flavonol adduct	1.03	526	M^+^	737.1710	737.1712	0.3	C_36_H_33_O_17_	737.1713 (100), 575.1177 (71), 557.1075 (24), 423.0707 (16), 329.0656 (59), 287.0548 (35)
2	4-*O*-caffeoylquinic acid * (cryptochlorogenic acid)	2.16	325	M−H^−^	353.0877	353.0878	0.3	C_16_H_17_O_9_	353.0875 (32), 191.0566 (56), 179.0350 (81), 173.0458 (100), 135.0453 (47)
3	5-*O*-caffeoylquinic acid * (chlorogenic acid)	2.38	325	2 M−H^−^	707.1825	707.1829	0.6	C_32_H_35_O_18_	707.1825 (92), 353.0877 (100), 191.0557 (91)
				M−H^−^	353.0879	353.0878	−0.3	C_16_H_17_O_9_	191.0561 (100)
4	4-*O*-*p*-coumaroylquinic acid **	3.96	312	2 M−H^−^	675.1930	675.1931	0.1	C_32_H_35_O_16_	337.0926 (100), 173.0451 (90)
				M−H^−^	337.0929	337.0929	−0.1	C_16_H_17_O_8_	173.0452 (100), 163.0396 (24)
5	cyanidin-3-*O*- galactoside * (ideain)	4.61	517	M^+^	449.1079	449.1078	−0.2	C_21_H_21_O_11_	287.0554 (100)
6	cyanidin-3-*O*- glucoside * (kuromanin)	5.37	519	M^+^	449.1076	449.1078	0.5	C_21_H_21_O_11_	287.0550 (100)
7a	5-carboxy-pyrano-cyanidin-hexoside	5.85	508	M^+^	517.0976	517.0977	0.1	C_24_H_21_O_13_	355.0450 (100)
7b	cyanidin-3-*O*- arabinoside *	5.85	508	M^+^	419.0972	419.0973	0.2	C_20_H_19_O_10_	287.0551 (100)
8	cyanidin-*O*- pentoside (1)	6.73	515	M^+^	419.0978	419.0973	−1.2	C_20_H_19_O_10_	287.0553 (100)
9	cyanidin-*O*- pentoside (2)	6.96	517	M^+^	419.0969	419.0973	0.9	C_20_H_19_O_10_	287.0551(100)
10	quercetin-3-*O*-galactoside * (hyperoside)	7.42	353	M−H^−^	463.088	463.0882	0.3	C_21_H_19_O_12_	463.0877 (100), 301.0344 (23), 300.0274 (61), 271.0246 (25), 255.0295 (14)
				M+H^+^	465.1029	465.1028	−0.2	C_21_H_21_O_12_	303.0499 (100)
11	cyanidin	7.50	524	M^+^	287.0550	287.055	0.0	C_15_H_11_O_6_	-
12a	quercetin-3-*O*- rutinoside * (rutin)	7.60	350	M−H^−^	609.1458	609.1461	0.5	C_27_H_29_O_16_	609.1465 (100), 301.0345 (24), 300.0280 (69), 271.0250 (8), 255.0292 (4)
				M+H^+^	611.1604	611.1607	0.4	C_27_H_31_O_16_	465.1031 (12), 303.0494 (100)
12b	quercetin-3-*O*- glucoside * (isoquercitrin)	7.66	352	M−H^−^	463.0881	463.0882	0.1	C_21_H_19_O_12_	463.0880 (100), 301.0345 (37), 300.0280 (63), 271.0251 (38), 255.0300 (23), 243.0302 (9)
				M+H^+^	465.1027	465.1028	0.1	C_21_H_21_O_12_	303.0497 (100)
13	phloretin-2′-*O*- xyloglucoside *	7.89	284	M−H^−^	567.1713	567.1719	1.1	C_26_H_31_O_14_	273.0770 (100), 167.0345 (83)
				M+H^+^	569.1865	569.1865	−0.1	C_26_H_33_O_14_	437.1441 (47), 275.0914 (100)
14	quercetin-3-*O*- xyloside * (reynutrin)	7.99	350	M−H^−^	433.0774	433.0776	0.6	C_20_H_17_O_11_	433.0771 (100), 301.0351 (51), 300.0278 (75), 271.0248 (47), 255.0295 (20), 243.0296 (13)
				M+H^+^	435.0918	435.0922	0.9	C_20_H_19_O_11_	303.0496 (100)
15	quercetin-3-*O*-arabinoside * (avicularin)	8.23	353	M−H^−^	433.0774	433.0776	0.5	C_20_H_17_O_11_	433.0778 (100), 301.0345 (25), 300.0279 (92), 271.0251 (52), 255.0304 (26), 243.0296 (16)
				M+H^+^	435.0924	435.0922	−0.4	C_20_H_19_O_11_	303.0499 (100)
16	phloretin-2′-*O*- glucoside * (phlorizin)	8.53	284	M−H^−^	435.1294	435.1297	0.7	C_21_H_23_O_10_	273.0769 (100), 167.0345 (18), 123.0454 (7)
				M+H^+^	437.1444	437.1442	−0.4	C_21_H_25_O_10_	275.0916 (100)
17	quercetin-3-*O*-rhamnoside * (quercitrin)	8.58	350	M−H^−^	447.0929	447.0933	0.8	C_21_H_19_O_11_	447.0930 (100), 301.0344 (52), 300.0278 (62), 271.0252 (42), 255.0301 (20), 243.0301 (12)
				M+H^+^	449.1076	449.1078	0.5	C_21_H_21_O_11_	303.0497 (100)

* Verified using authentic reference standards. ** Tentatively identified.

**Table 3:** Levels of anthocyanins and colorless (poly)phenols in raw juice derived of dejuicing red-fleshed apples with three different pressing systems in 2019 (*n* = 2 technological replicates) and 2020 (*n* = 2).

	2019				2020	
Analytes [mg/L]	Spiral Filter Press	Horizotal Filter Press	Decanter	Spiral Filter Press	Horizotal Filter Press	Decanter
*anthocyanins*						
anthocyanin-flavonol adduct	4.95 ± 0.14 ^a^	1.94 ± 0.00 ^b^	1.83 ± 0.00 ^b^	6.33 ± 0.01 ^a^	2.11 ± 0.00 ^b^	2.04 ± 0.07 ^b^
cyanidin-3-*O*-galactoside	30.51 ± 0.22 ^a^	5.16 ± 0.23 ^b^	3.54 ± 0.21 ^b^	53.52 ± 0.77 ^a^	7.35 ± 0.10 ^b^	7.47 ± 2.14 ^b^
cyanidin-3-*O*-glucoside	1.74 ± 0.01 ^a^	1.43 ± 0.00 ^b^	1.44 ± 0.01 ^b^	1.97 ± 0.01 ^a^	1.42 ± 0.00 ^b^	1.43 ± 0.01 ^b^
5-carboxy-pyrano-cyanidin-hexoside	1.95 ± 0.00 ^a^	1.37 ± 0.02 ^b^	1.33 ± 0.00 ^c^	2.31 ± 0.01 ^a^	1.4 ± 0.00 ^b^	1.39 ± 0.03 ^b^
cyanidin-3-*O*-arabinoside	2.18 ± 0.02 ^a^	1.73 ± 0.04 ^b^	1.70 ± 0.02 ^b^	2.50 ± 0.01 ^a^	1.92 ± 0.00 ^b^	1.83 ± 0.05 ^b^
cyanidin-*O*-pentoside (1)	1.81 ± 0.01 ^a^	1.43 ± 0.05 ^b^	1.32 ± 0.00 ^b^	2.03 ± 0.00 ^a^	1.37 ± 0.01 ^b^	1.37 ± 0.03 ^b^
cyanidin-*O*-pentoside (2)	3.90 ± 0.05 ^a^	1.50 ± 0.02 ^b^	1.43 ± 0.01 ^b^	5.40 ± 0.01 ^a^	1.70 ± 0.01 ^b^	1.67 ± 0.14 ^b^
cyanidin	0.84 ± 0.00	n. d.	n. d.	0.86 ± 0.00	n. d.	n. d.
total anthocyanins	47.89 ± 0.06 ^a^	14.61 ± 0.04 ^b^	12.59 ± 0.04 ^b^	74.91 ± 0.10 ^a^	17.26 ± 0.02 ^b^	17.19 ± 0.35 ^b^
*colorless (poly)phenols*						
4-*O*-caffeoylquinic acid	2.63 ± 0.24 ^b^	4.99 ± 0.08 ^a^	4.88 ± 0.09 ^a^	3.90 ± 0.10 ^b^	10.65 ± 0.28 ^a^	9.84 ± 0.25 ^a^
5-*O*-caffeoylquinic acid	81.87 ± 4.28 ^a^	30.23 ± 1.30 ^b^	31.00 ± 0.38 ^b^	150.62 ± 1.54 ^a^	49.17 ± 1.17 ^b^	57.34 ± 4.15 ^b^
4-*O*-*p*-coumaroylquinic acid	26.09 ± 0.90 ^a^	12.50 ± 0.58 ^b^	14.46 ± 0.12 ^b^	62.49 ± 0.45 ^a^	30.15 ± 0.29 ^b^	38.75 ± 1.22 ^b^
quercetin-3-*O*-galactoside	2.92 ± 0.07 ^a^	2.73 ± 0.01 ^a^	2.01 ± 0.01 ^b^	4.23 ± 0.23	4.02 ± 0.01	2.73 ± 0.07
quercetin-3-*O*-glucoside	1.27 ± 0.02 ^a^	1.10 ± 0.01 ^ab^	0.91 ± 0.01 ^b^	1.50 ± 0.09	1.55 ± 0.01	1.11 ± 0.02
phloretin-2′-*O*-xyloglucoside	40.16 ± 0.41 ^a^	13.54 ± 0.40 ^b^	17.08 ± 0.13 ^b^	73.43 ± 0.41 ^a^	26.36 ± 0.12 ^b^	34.91 ± 0.94 ^b^
quercetin-3-*O*-xyloside	0.88 ± 0.01 ^a^	0.87 ± 0.01 ^a^	0.73 ± 0.00 ^b^	1.18 ± 0.05	1.12 ± 0.01	0.89 ± 0.01
quercetin-3-*O*-arabinoside	1.23 ± 0.02 ^a^	1.22 ± 0.01 ^a^	0.95 ± 0.00 ^b^	1.62 ± 0.08	1.66 ± 0.03	1.20 ± 0.02
phloretin-2′-*O*-glucoside	10.84 ± 0.19 ^a^	5.49 ± 0.11 ^b^	4.87 ± 0.03 ^b^	21.74 ± 0.85 ^a^	12.01 ± 0.03 ^ab^	10.65 ± 0.32 ^b^
quercetin-3-*O*-rhamnoside	1.72 ± 0.01 ^a^	1.39 ± 0.02 ^b^	1.15 ± 0.01 ^b^	2.83 ± 0.09	2.21 ± 0.00	1.88 ± 0.05
total colorless (poly)phenols	169.60 ± 0.61 ^a^	74.06 ± 0.25 ^b^	78.04 ± 0.08 ^b^	323.53 ± 0.48 ^a^	13.89 ± 0.36 ^b^	15.93 ± 1.28 ^b^

Different superscript letters (a, b) indicate significant (*p* < 0.05) differences of means within one year. n. d.: not detected.

## Data Availability

Not applicable.

## Supplementary Materials

The following supporting information can be downloaded at: https://www.mdpi.com/article/10.3390/molecules27082459/s1, Table S1. Oxygen contents in juices derived from three pressing systems during 24 weeks of storage at three temperatures (4, 20, and 37 °C) in 2020; Table S2. Chromameter CIE-L*a*b* values of cloudy raw juice derived of dejuicing red-fleshed apples with three different pressing systems in 2019 (*n* = 2, technological replicates) and 2020 (*n* = 2); Table S3. Levels of colorless (poly)phenols in juices derived from three pressing systems in 2020 during 24 weeks of storage at 4 °C; Table S4. Levels of colorless (poly)phenols in juices derived from three pressing systems in 2020 during 24 weeks of storage at 37 °C. Table S5. Antioxidant capacity (TEAC assay) in juices derived from three pressing systems during 24 weeks of storage at two temperatures (4 and 20 °C) in 2020.
