# Supplementary material for: Comparative Evaluation of Juices from Red-Fleshed Apples after Production with Different Dejuicing Systems and Subsequent Storage
Source: Molecules. 2022 Apr 11;27(8):2459. doi: 10.3390/molecules27082459 (PMC9028955; doi:10.3390/molecules27082459)
Supplement: Supplementary file 1 [file molecules-27-02459-s001.zip › molecules-1641608-supplementary.pdf]

molecules
MDPI

Supplementary Materials

Comparative Evaluation of Juices from Red-Fleshed Apples after Production with Different Dejuicing Systems and Subsequent Storage

Annette Wagner [1], Stefan Dussling [1], Stefano Scansani [2], Peter Bach [1], Michael Ludwig [1], Christof B. Steingass [1], Frank Will [1] and Ralf Schweiggert [1,*]

1 Workgroup Analysis and Technology of Plant-based Foods, Department of Beverage Research, Hochschule Geisenheim University, Von-Lade-Strasse 1, 65366 Geisenheim, Germany
2 Department of Microbiology and Biochemistry, Hochschule Geisenheim University, Von-Lade-Strasse 1, 65366 Geisenheim, Germany

* Correspondence: ralf.schweiggert@hs-gm.de; Tel.: +49-6722 -502-311; Fax: +49-6722-502-310

Table S1. Oxygen contents in juices derived from three pressing systems during 24 weeks of storage at three temperatures (4, 20, and 37°C) in 2020. Data represent means and standard deviations of two technological replicates.

		Week						
		0	2	4	6	12	16	24
4°C	spiral press	6.56 ± 0.9	1.43 ± 0.49	0.64 ± 0.08	0.23 ± 0.08	0.22 ± 0.05	0.23 ± 0.05	0.25 ± 0.00
	horizontal press	6.80 ± 0.3	1.31 ± 0.62	0.45 ± 0.18	0.22 ± 0.01	0.14 ± 0.01	0.15 ± 0.03	0.19 ± 0.02
	decanter	7.28 ± 0.3	1.00 ± 0.21	0.51 ± 0.08	0.33 ± 0.01	0.37 ± 0.16	0.23 ± 0.14	0.25 ± 0.06
20°C	spiral press	6.56 ± 0.9	0.83 ± 0.00	0.40 ± 0.13	0.26 ± 0.09	0.13 ± 0.00	0.12 ± 0.01	0.18 ± 0.08
	horizontal press	6.80 ± 0.3	0.95 ± 0.03	0.37 ± 0.10	0.30 ± 0.06	0.11 ± 0.01	0.22 ± 0.17	0.09 ± 0.05
	decanter	7.28 ± 0.3	0.95 ± 0.04	0.60 ± 0.24	0.34 ± 0.02	0.15 ± 0.01	0.16 ± 0.00	0.14 ± 0.08
37°C	spiral press	6.56 ± 0.9	0.92 ± 0.05	0.40 ± 0.04	0.19 ± 0.04	0.12 ± 0.01	0.14 ± 0.01	0.22 ± 0.07
	horizontal press	6.80 ± 0.3	0.88 ± 0.01	0.40 ± 0.13	0.21 ± 0.04	0.11 ± 0.01	0.09 ± 0.01	0.19 ± 0.08
	decanter	7.28 ± 0.3	1.20 ± 0.40	0.46 ± 0.21	0.22 ± 0.09	0.11 ± 0.00	0.10 ± 0.01	0.27 ± 0.00

Table S2. Chromameter CIE-L*a*b* values of cloudy raw juice derived of dejuicing red-fleshed apples with three different pressing systems in 2019 (n=2, technological replicates) and 2020 (*n*=2).

	**2019**			**2020**		
	**Spiral filter press**	**Horizotal filter press**	**Decanter**	**Spiral filter press**	**Horizotal filter press**	**Decanter**
CIE-L*a*b*						
L*	36.5 ± 0.1^{a}	31.2 ± 0.3^{c}	33 ± 0.1^{b}	35.1 ± 0.7^{a}	30.2 ± 0.3^{c}	32.6 ± 0.1^{b}
a*	17.5 ± 0.9^{a}	5.3 ± 0.1^{b}	4.9 ± 0.2^{b}	18.4 ± 1^{a}	4.7 ± 0.3^{b}	6.1 ± 1.8^{b}
b*	3.8 ± 0.4	2.4 ± 0.2	3.2 ± 0.6	5.1 ± 0.3^{a}	1.6 ± 0.1^{b}	2.9 ± 0.7^{b}
hue angle h°	12.2 ± 0.6^{ab}	24.3 ± 1.7^{b}	32.5 ± 6.4^{a}	15.3 ± 0.1	19.4 ± 2.3	27 ± 12.1
chroma C*	17.9 ± 0.9^{a}	5.8 ± 0^{b}	5.9 ± 0.2^{b}	19.1 ± 1.1^{a}	4.9 ± 0.2^{b}	6.8 ± 1.4^{b}

Different superscript letters indicate significant ($p < 0.05$) differences of means within one year.

Table S3. Levels of colorless (poly)phenols in juices derived from three pressing systems in 2020 during 24 weeks of storage at 4°C. Data represent means and standard deviations of two technological replicates.

		**Week**						
		**0**	**4**	**8**	**12**	**16**	**20**	**24**
	spiral filter press	73.4 ± 0.4	71.2 ± 0.3	71.9 ± 0.5	72.3 ± 0.5	70.9 ± 0.4	71.2 ± 0.3	69 ± 0^{a}
phloretin-2′-*O*-xyloglucoside	horizontal filter press	26.4 ± 0.1	25 ± 0.1	24.7 ± 0	24.7 ± 0.3	21.7 ± 0.1	17.8 ± 0.1	23.6 ± 0.2^{b}
	decanter	34.9 ± 0.9	32.8 ± 1	32.7 ± 1	27.6 ± 0	26.3 ± 0.5	31.3 ± 0.9	30.9 ± 0.9^{b}
	spiral filter press	21.7 ± 0.9	20.8 ± 0.8	20.3 ± 0.9	21.5 ± 0.9	21.2 ± 0.9	21.1 ± 0.8	18.1 ± 0^{a}
phloretin-2′-*O*-glucoside	horizontal filter press	12 ± 0	11.7 ± 0.1	11.2 ± 0.2	11.8 ± 0	10.5 ± 0.1	8.5 ± 0.1	11.5 ± 0^{b}
	decanter	10.6 ± 0.3	9.9 ± 0.3	10.1 ± 0.3	9.1 ± 0	8.6 ± 0.1	10.1 ± 0.3	9.9 ± 0.3^{b}
	spiral filter press	3.9 ± 0.1	3.5 ± 0	3.7 ± 0	3.4 ± 0.1	3.5 ± 0.1	3.8 ± 0	3.5 ± 0^{b}
4-*O*-caffeoylquinic acid	horizontal filter press	10.6 ± 0.3	9.8 ± 0.1	11.2 ± 0.3	11.3 ± 0	10.1 ± 0.2	8.2 ± 0.3	11.5 ± 0^{a}
	decanter	9.8 ± 0.3	8.9 ± 0.4	11.4 ± 0	11.4 ± 0	9.9 ± 0	11.3 ± 0	11.2 ± 0.2^{a}
	spiral filter press	150.6 ± 1.5	143.8 ± 1.6	142.3 ± 2.3	143.2 ± 2.3	141.6 ± 1.4	138.7 ± 0.1	142.8 ± 1.8^{a}
5-*O*-caffeoylquinic acid	horizontal filter press	49.2 ± 1.2	45.3 ± 1.2	44.1 ± 0.1	45 ± 1.6	40.4 ± 0.6	33.2 ± 0.1	46 ± 1.2^{b}
	decanter	57.3 ± 4.2	52.5 ± 4.4	53.3 ± 4.2	43.7 ± 0.1	43.7 ± 2.2	53.1 ± 4	53.2 ± 4.6^{b}
	spiral filter press	62.5 ± 0.5	60.5 ± 0.5	55.8 ± 0.2	59.1 ± 0.2	58.3 ± 0.4	60.1 ± 0	59.3 ± 0.5^{a}
4-*O*-*p*-coumaroylquinic	horizontal filter press	30.2 ± 0.3	28.5 ± 0.3	25 ± 0.7	27.7 ± 0.1	24.7 ± 0.4	20.7 ± 0.7	28.2 ± 0.3^{b}
	decanter	38.7 ± 1.2	37.2 ± 1.4	34.3 ± 1.4	33.3 ± 0	31.5 ± 0.7	36.5 ± 1.3	36.7 ± 1.5^{b}
	spiral filter press	4.2 ± 0.2	4 ± 0.2	3.9 ± 0.2	4 ± 0.2	3.9 ± 0.2	3.3 ± 0	4 ± 0.2
quercetin-3-*O*-galactoside	horizontal filter press	4 ± 0	3.8 ± 0	3.6 ± 0.1	3.8 ± 0	3.4 ± 0	2.9 ± 0	3.7 ± 0
	decanter	2.7 ± 0.1	2.6 ± 0.1	2.6 ± 0.1	2.4 ± 0	2.3 ± 0	2.5 ± 0.1	2.6 ± 0.1
	spiral filter press	1.6 ± 0.1	1.6 ± 0.1	1.6 ± 0.1	1.6 ± 0.1	1.6 ± 0.1	1.4 ± 0	1.6 ± 0.1
quercetin-3-*O*-arabinoside	horizontal filter press	1.7 ± 0	1.7 ± 0	1.6 ± 0	1.6 ± 0	1.5 ± 0	1.3 ± 0	1.6 ± 0
	decanter	1.2 ± 0	1.2 ± 0	1.2 ± 0	1.1 ± 0	1 ± 0	1.1 ± 0	1.2 ± 0
	spiral filter press	2.8 ± 0.1	2.7 ± 0.1	2.7 ± 0.1	2.8 ± 0.1	2.7 ± 0.1	2.4 ± 0	2.8 ± 0.1
quercetin-3-*O*-rhamnoside	horizontal filter press	2.2 ± 0	2.1 ± 0	2 ± 0	2.2 ± 0	1.9 ± 0	1.7 ± 0	2.1 ± 0
	decanter	1.9 ± 0	1.8 ± 0	1.8 ± 0	1.7 ± 0	1.6 ± 0	1.8 ± 0	1.8 ± 0
	spiral filter press	323.5 ± 0.5	310.9 ± 0.5	305 ± 0.7	310.5 ± 0.7	306.4 ± 0.5	304.3 ± 0.3	303.5 ± 0.6^{a}

total colorless (poly)phenols	horizontal filter press	138.9 ± 0.4	130.6 ± 0.4	125.9 ± 0.2	130.6 ± 0.5	116.4 ± 0.2	96.5 ± 0.2	130.5 ± 0.4[b]
	decanter	159.3 ± 1.3	148.8 ± 1.4	149.4 ± 1.3	132.1 ± 0	126.6 ± 0.7	149.5 ± 1.3	149.2 ± 1.4[b]

Different superscript letters indicate significant (p < 0.05) differences of means between the pressing systems for the single colorless (poly)phenols.

Table S4. Levels of colorless (poly)phenols in juices derived from three pressing systems in 2020 during 24 weeks of storage at 37°C. Data represent means and standard deviations of two technological replicates.

		**Week**						
		**0**	**4**	**8**	**12**	**16**	**20**	**24**
	spiral filter press	73.4 ± 0.4	69.1 ± 0.5	68.3 ± 0.6	67.1 ± 0.4	64.1 ± 0.1	49.1 ± 0.5	58 ± 0[a]
phloretin-2′-*O*-xyloglucoside	horizontal filter press	26.4 ± 0.1	24.5 ± 0.2	28.6 ± 0.9	23.4 ± 0.1	19.1 ± 0.4	21.6 ± 0.1	20.7 ± 0.1[b]
	decanter	34.9 ± 0.9	31.2 ± 0.8	30 ± 0.7	30.4 ± 0.7	27 ± 0.9	24.2 ± 0.9	28 ± 0.9[b]
	spiral filter press	21.7 ± 0.9	20.8 ± 0.8	20.5 ± 1	21 ± 1	20.7 ± 0.9	16 ± 0.4	19.3 ± 0.9
phloretin-2′-*O*-glucoside	horizontal filter press	12 ± 0	11.6 ± 0	10.9 ± 0.1	11.7 ± 0	9.9 ± 0.3	11.4 ± 0.1	11.1 ± 0.1
	decanter	10.6 ± 0.3	10.1 ± 0.2	9.9 ± 0.2	10.2 ± 0.3	9.3 ± 0.4	8.5 ± 0.4	9.6 ± 0.3
	spiral filter press	3.9 ± 0.1	3.6 ± 0	3.6 ± 0	4.1 ± 0	4.3 ± 0	3.4 ± 0.2	4.6 ± 0.1[b]
4-*O*-caffeoylquinic acid	horizontal filter press	10.6 ± 0.3	9.1 ± 0	9.6 ± 0.3	9.3 ± 0	7.3 ± 0.2	8.3 ± 0	7.8 ± 0[a]
	decanter	9.8 ± 0.3	7.8 ± 0.4	9.3 ± 0	9.1 ± 0	7.8 ± 0.2	6.9 ± 0.4	7.7 ± 0[a]
	spiral filter press	150.6 ± 1.5	135.1 ± 1	125.3 ± 2.7	122.3 ± 1.8	117 ± 1.1	92.7 ± 2.3	110.9 ± 1.4[a]
5-*O*-caffeoylquinic acid	horizontal filter press	49.2 ± 1.2	42.4 ± 1.3	40 ± 0.4	40.5 ± 0.7	33.1 ± 2	37.9 ± 1	37.2 ± 0.9[b]
	decanter	57.3 ± 4.2	48.1 ± 3.5	45.8 ± 3	46.6 ± 3.3	41.6 ± 3.8	37.6 ± 3.9	44.3 ± 3.8[b]
	spiral filter press	62.5 ± 0.5	56 ± 0.4	47.7 ± 0.1	46.8 ± 0.1	43.6 ± 0.3	33.4 ± 1.4	38.4 ± 0.3[a]
4-*O*-*p*-coumaroylquinic	horizontal filter press	30.2 ± 0.3	26.5 ± 0.2	21.9 ± 0.4	22.3 ± 0.3	17.7 ± 0.5	19.4 ± 0.1	18.5 ± 0.2[b]
	decanter	38.7 ± 1.2	33.5 ± 1	28.3 ± 0.8	29.1 ± 0.8	25.1 ± 1.3	21.6 ± 1.3	24.5 ± 0.9[b]
	spiral filter press	4.2 ± 0.2	3.7 ± 0.2	3.2 ± 0.2	3 ± 0.2	2.8 ± 0.2	2 ± 0.1	2.2 ± 0.1
quercetin-3-*O*-galactoside	horizontal filter press	4 ± 0	3.6 ± 0	3.1 ± 0	3 ± 0	2.4 ± 0	2.5 ± 0	2.4 ± 0
	decanter	2.7 ± 0.1	2.4 ± 0.1	2.1 ± 0	2.1 ± 0	1.8 ± 0.1	1.6 ± 0.1	1.6 ± 0
	spiral filter press	1.6 ± 0.1	1.1 ± 0.1	0.8 ± 0	0.6 ± 0	0.8 ± 0.1	0.5 ± 0	0.5 ± 0
quercetin-3-*O*-arabinoside	horizontal filter press	1.7 ± 0	1.2 ± 0	0.9 ± 0	0.7 ± 0	0.6 ± 0	0.5 ± 0	0.5 ± 0
	decanter	1.2 ± 0	0.8 ± 0	0.7 ± 0	0.6 ± 0	0.5 ± 0	0.5 ± 0	0.5 ± 0
	spiral filter press	2.8 ± 0.1	2.5 ± 0.1	2.2 ± 0.1	2.1 ± 0.1	1.9 ± 0.1	1.5 ± 0	1.6 ± 0.1
quercetin-3-*O*-rhamnoside	horizontal filter press	2.2 ± 0	2.1 ± 0	1.8 ± 0	1.8 ± 0	1.5 ± 0	1.6 ± 0	1.5 ± 0
	decanter	1.9 ± 0	1.8 ± 0	1.5 ± 0	1.5 ± 0	1.3 ± 0	1.2 ± 0	1.3 ± 0
	spiral filter press	316.8 ± 0.5	294.3 ± 0.4	274 ± 0.8	269 ± 0.6	257.1 ± 0.4	200.1 ± 0.8	237 ± 0.5[a]
total colorless (poly)phenols	horizontal filter press	132.3 ± 0.4	123.3 ± 0.4	119 ± 0.3	114.6 ± 0.2	93.3 ± 0.6	105.1 ± 0.3	101.4 ± 0.3[b]
	decanter	155.1 ± 1.5	137.5 ± 1.1	129.3 ± 0.9	131.1 ± 1	115.8 ± 1.2	103.4 ± 1.2	118.7 ± 1.2[b]

Different superscript letters indicate significant (p < 0.05) differences of means between the pressing systems for the single colorless (poly)phenols.

Table S5. Antioxidant capacity (TEAC assay) in juices derived from three pressing systems during 24 weeks of storage at two temperatures (4 and 20°C) in 2020. Data represent means and standard deviations of two technological replicates.

		Week		
		**0**	**12**	**24**
4°C	spiral press	6.52 ± 0.68	5.43 ± 0.45	6.08 ± 1.10[a]
	horizontal press	3.55 ± 0.48	2.90 ± 0.17	2.99 ± 0.90[b]
	decanter	3.92 ± 0.63	3.21 ± 0.48	3.64 ± 0.95[ab]
20°C	spiral press	6.52 ± 0.68	4.61 ± 0.99	6.04 ± 1.01[a]
	horizontal press	3.55 ± 0.48	2.89 ± 0.30	2.92 ± 0.64[b]
	decanter	3.92 ± 0.48	3.27 ± 0.32	3.53 ± 3.53[b]

Different superscript letters indicate significant ($p < 0.05$) differences of means between the pressing systems within one storage temperature.
